# Smoke-free hospitals – the English experience: results from a survey, interviews, and site visits

**DOI:** 10.1186/1472-6963-8-41

**Published:** 2008-02-18

**Authors:** Elena Ratschen, John Britton, Ann McNeill

**Affiliations:** 1 University of Nottingham, Clinical Sciences Building, City Hospital, Nottingham NG5 1PB, UK

## Abstract

**Background:**

According to the provisions of the Health Act 2006, NHS acute Trusts had to become smoke-free by July 2007. Mental health Trusts were granted a further year before all indoor smoking areas have to be removed. This study was carried out to determine the extent of smoke-free policy implementation in English NHS acute and mental health Trusts, and to explore challenges and impacts related to policy implementation.

**Methods:**

Questionnaire-based survey of all English NHS acute and mental health hospital settings, supplemented by semi-structured telephone interviews with 22 respondents and direct observation at a sample of 15 Trusts (22 different sites). Human Resources Directors of all 245 English NHS Trusts providing acute and/or mental health inpatient care were identified as potential study participants. Main outcome measures comprised the proportions of Trusts reporting smoke-free policy implementation; whether these relate to buildings only or to whole premises including grounds; most frequently reported exemptions; reported and observed frequencies of policy breaches.

**Results:**

Smoke-free policies were reported to be implemented in all mental health and 98% of acute settings studied. They applied to whole premises including grounds in 84% of acute, and 64% of mental health settings. However, exemptions were granted by 50% of acute and 78% of mental health settings, typically for bereaved relatives or psychiatric patients, in sheltered outdoor areas and smoking rooms. Reported challenges included policy enforcement and related risks of abuse, and litter on premises and adjacent public grounds. Nearly two thirds of acute and over a third of mental health trusts reported that policy infringements occurred on a daily basis. Indeed, patients and visitors were observed smoking at 94% of acute sites visited and staff smoking at 35% of them.

**Conclusion:**

NHS hospitals should play an exemplary role in making a smoke-free environment the norm. Although *s*moke-free policies have been implemented in nearly all English NHS hospitals, exemptions are frequently granted and policy breaches appear to be commonplace.

## Background

The prevention of exposure to environmental tobacco smoke and the promotion of smoking cessation are central components of the international WHO Framework Convention on Tobacco Control [1]. In recent years, legal provisions for smoke-free policies in workplaces and public places have been increasingly introduced in many countries worldwide. In England, the *Health Act 2006 *[2] established the legal framework relating to smoke-free policies in work and public places.

The dangers of passive smoking and the need to lead by example in promoting health and preventing disease, have resulted in health services often being at the forefront of introducing smoke-free policies. In England, since the 1980s, several edicts have required National Health Service (NHS) hospitals to implement smoke-free policies [3,4].

Studies indicate that the implementation of smoke-free policies in both acute and mental health hospital settings is achievable [5-8] with potentially beneficial impacts on aspects such as exposure to environmental tobacco smoke[9] and smoking prevalence among staff[10,11]. However, it has also been found that the enforcement of smoke-free policies in health services remains a challenge after implementation and a national survey of smoke-free policies in the NHS in 2003 highlighted that many health services were not smoke-free and identified several problems and barriers in achieving this [12,13]. In 2005, the Health Development Agency (HDA) therefore published new guidance[13] to support Trusts' efforts in comprehensive policy implementation. The guidance established that a complete smoke-free policy covering buildings and grounds without allowing for exemptions was a "gold standard" [13] for hospitals as health promoting organisations. A further pronouncement was then made by government requesting the health service to be smokefree by the end of 2006[14].

This study aimed to investigate the extent of and experience with smoke-free policy implementation in English NHS acute and mental health settings shortly after that deadline in February 2007. The Health Act makes it compulsory for all hospitals to be smoke-free after 1 July 2007 but allows mental health Trusts to keep smoking rooms under certain conditions until July 2008 [15].

## Methods

### Study Institutions and Participants

A list of all English NHS Trusts providing acute and/or mental health services in inpatient facilities was purchased from the data provider *Binley's – Health & Care Information Specialist*, cooperating partner of the NHS confederation. A total of 245 Trusts were identified (Acute Trusts = 173, mental health settings = 72), four of which were later excluded when questionnaire responses revealed that no inpatient treatment was provided. As previous studies suggested that members of Human Resources Departments are involved in the development of smoke-free policies more often than any other staff group[12], Human Resources Directors of the Trusts were identified as potential study participants. Where no Human Resources Director or alternative main personnel contact could be identified, Chief Executives were chosen instead (n = 9). Ethical approval for the study was obtained from the local Research Ethics Committee in February 2007.

### Outcome Measures

Primary outcome measures included: the proportion of Trusts reporting to have implemented smoke-free policies; the proportion of Trusts whose policies referred to the buildings only, or the whole premises including grounds; most frequently granted exemptions to smoke-free policies. Secondary outcome measures comprised: the frequency of reported and observed policy breaches; information referring to selected aspects of policy development; most frequently named success factors, challenges, and impacts related to policy implementation.

### Study Instruments

The questionnaire was designed to collect structured basic information on smoke-free policy implementation on the basis of a questionnaire used in previous research by the HDA[12], and further relevant aspects identified from the *Guidance for smoke-free hospital trusts*[13]. It was issued to potential participants of all 245 Trusts by post and additionally made accessible for online completion in February 2007. Two reminder letters were sent to non-respondents after three and six weeks. A formal request of information under the Environmental Information Regulations [EIR][16] was made after ten weeks. Trusts were asked to provide specified basic data on their policies in the course of 20 working days, or to complete the study questionnaire.

Telephone interviews were carried out to supplement the information provided in the questionnaires. A 30% systematic sample of questionnaire respondents who had indicated their availability for an interview in the questionnaire was drawn from respondents as listed according to the order in which questionnaire responses had been recorded, starting at a random number between one and five, and then one and ten respectively. A semi-structured interview guide of pre-defined and emerging categories was developed for the interviews that lasted around 30 minutes.

Site visits were carried out to investigate visible indicators of smoking at a convenience sample of 22 Trust sites, and to validate information obtained from the questionnaires. A checklist including categories on signage, observed smoking behaviour, litter on grounds and information on the smoke-free policy was used to record the information. Pictures were taken to document the observations.

Where appropriate, triangulation of data was used for cross-checks to determine the validity of the information collected.

### Analysis

Questionnaire responses were coded and entered into an SPSS (v.14.0) database to generate the outcome measures. Since the study did not use a sampling frame but included all eligible Trusts, no statistical measures of effect were obtained. Free text comments were summarised according to recurring themes. Telephone interviews were tape-recorded and responses allocated to the appropriate response category of the interview guide. Information from site visits was recorded in a checklist.

## Results

### Questionnaires

Questionnaires were returned by 77% (186) of Trusts, 76% of which were acute Trusts and 79% mental health settings. There was evidence from free text comments and email addresses provided that the Directors had partly delegated study participation to Assistant Directors or staff members in charge of smoke-free policy. Sixty per cent (145) of Trusts had responded to the first letter and two follow-ups, and 17% (41) completed the questionnaire in response to the EIR request. From those hospitals that did not send back a questionnaire, two per cent (4) provided basic information in response to the EIR request, which allowed details on the characteristics of smoke-free policies (partial/complete) and exemptions granted to be included in the information given below. The EIR request was not answered by 21% (51) Trusts.

Ninety eight per cent of respondents reported that smoke-free policies were implemented in their Trusts. The remaining two per cent had set dates for implementation before July 2007. In acute Trusts, 84% of the policies applied to the whole premises including grounds, and 16% to all buildings. For mental health settings, 64% reported policies that applied to the whole premises, 29% to all buildings, and 7% to parts of the buildings. Policies that applied to the whole premises including grounds and not allowing for any exemptions were reported by 41% of acute and 13% of mental health settings, amounting to 33% of all respondents.

Half of the acute (50%) and 78% of mental health settings reported allowing exemptions to the policies. In acute Trusts that allowed for exemptions, these were granted most frequently for bereaved/distressed relatives (45%), and in sheltered outdoor areas (25%). The provision of smoking rooms was reported by seven (6%) of acute Trusts, indicating non-compliance with the Health Act 2006 at the time of questionnaire completion. Exceptions for psychiatric patients were made in 15% of acute and 65% of mental health settings that reported allowing exemptions. Smoking rooms were provided in 42% of these mental health settings. NHS stop smoking services were reported to be advertised in 92% of acute and 80% of mental health settings.

Of respondents from acute Trusts, 65% stated that policy infringements occurred at least daily, as did 37% of respondents from mental health settings. However, 92% of respondents rated their policy as "quite successful" (66%) or "very successful" (26%). Table 1 gives further details of policy infringements and other sample characteristics.

**Table 1:** Questionnaire responses on smoke-free policy

**Question**	**Responses**	**Acute Trusts (%)**	**Mental Health Settings (%)**	**All Trusts (%)**
Trust Type/Setting	Acute	132 (76.3)**		186 (77.2)
	Mental Health*		54 (79.4)**	
Trust comprises more than one site	Yes	91 (68.9)	54 (100)	145 (78.0)
Frequency of infringements of policy	At least daily	86 (65.2)	20 (37.0)	106 (57.0)
	At least weekly	23 (17.4)	7 (13.0)	30 (16.1)
	At least monthly	4 (3.0)	2 (3.7)	6 (3.2)
	Less than monthly	7 (5.3)	6 (11.1)	13 (7.0)
	Never	0 (0.0)	2 (3.7)	2 (1.1)
	Don't know/not answered	12 (9.1)	17 (31.5)	29 (15.6)
Specific exemptions granted at sites which allow for exemptions (n = 186 plus 4 EIR responses in a form other than the questionnaire)	Bereaved/distressed relatives	30 (44.8)	4 (9.3)	34 (30.9)
	Smoking rooms	4 (6.0)	18 (41.9)	22 (20.0)
	Sheltered areas in grounds	17 (25.4)	17 (39.5)	34 (30.5)
	Psychiatric patients	10 (14.9)	28 (65.1)	38 (34.5)
	Entrances	2 (3.0)	2 (4.7)	4 (3.6)
	Long-stay patients	5 (7.5)	14 (32.6)	19 (17.3)
	Other	17 (25.4)	3 (7.0)	20 (18.2)
	Case-by-case basis°	9 (13.4)	3 (7.0)	12 (10.9)
	Terminally ill patients°	5 (7.5)	-	5 (4.5)
Overall success of policy implementation	Very successful	36 (27.3)	12 (22.2)	48 (25.8)
	Quite successful	85 (64.4)	38 (70.4)	123 (66.1)
	Not successful	5 (3.8)	2 (3.7)	7 (3.8)
	Don't know	6 (4.5)	2 (3.7)	8 (4.3)

* Comprise mental health, partnership, care and primary care trusts according to listings

** Percentages refer to total number of applicable Trust type: acute (n = 173), or mental health (n = 68)

° As specified in the context of the chosen option "Other"

A specific budget for smoke-free policy implementation had been available in 24% of the acute and 19% of mental health settings. The HDA guidance was reported to have been used during the drafting process by 79% and 85% of acute and mental health settings respectively. Almost 75% of respondents reported to have informed staff of the smoke-free policy comprehensively by disseminating information in meetings or special events and through at least two other ways of communication such as emails, newsletters, or the Trust intranet.

### Interviews

By the end of April 2007, 83 survey respondents had indicated their availability for a telephone interview. A 30% sample (25 Trusts) was taken, of which 22 agreed to participate and were interviewed after obtaining informed consent. All respondents rated the implementation of smoke-free policy as generally positive, though 59% highlighted related challenges. Several respondents (21%) said they had encountered fewer difficulties than anticipated.

#### Challenges: enforcement and adverse implications

The active involvement of all staff members was named as central to policy enforcement by 68% of respondents. However, the same number stated concerns regarding aggression and abuse, when challenging patients and visitors who smoked onsite, to explain the reluctance of staff to engage actively in enforcement. Difficulties in sustaining policy enforcement in certain areas, such as entrances and A&E departments, were mentioned by 68% of respondents. Half of the respondents reported that they allowed for exemptions on a case-by-case basis (although only two (9%) had indicated this in the questionnaire) but mentioned that discretion was applied to prevent passive exposure of third parties to tobacco smoke.

Staff, patients and visitors "congregating" in front of Trust premises to smoke, and related adverse effects on Trust image and environment were perceived as challenging by 64% of those interviewed. A further concern mentioned was that staff who smoked sometimes left the premises to smoke, outside their official break times. More than half (55%) described litter from cigarette ends on Trust premises as a problem.

#### Support for patients and staff

Nicotine Replacement Therapy (NRT) for patients was reported to be available in the hospital pharmacy by 77% of respondents. Onsite cessation support for patients was offered by 73% of Trusts, and all Trusts reported close collaboration with the NHS Stop Smoking Services. Assessments of patients' smoking status on admission were undertaken in 45% of all Trusts, and 9% (all acute Trusts) carried out pre-assessments of elective patients.

Staff were reported to be supported in smoking cessation through the provision of free or reduced NRT in 55% of Trusts. Almost all Trusts (95%) reported that they offered smoking cessation classes through occupational health and community services. Some respondents reported that the offers were not taken up well by staff and had therefore been reduced.

#### Determinants of success

Extensive communication and promotion of the smoke-free policy and its constant reinforcement were regarded as crucial for policy success by 77% of respondents. Some commented on the shortage of resources to achieve these aims. The rigorous banning of smoking from premises without exemptions was regarded as critical for successful policy implementation by 23% of interviewees. Over half (55%) of the respondents believed that a changed attitude towards smoking in public places after July 2007 would facilitate enforcement in the future.

#### Impact

More than half (55%) of respondents reported reduced exposure to environmental tobacco smoke in the buildings and less smoking in the grounds as a result of the smoke-free policy. Anecdotal evidence for a reduction in smoking prevalence amongst staff following policy implementation was reported by 59% of respondents. Moreover, 41% believed that enhanced support with regard to smoking cessation might add to patients' motivation to stop smoking. Several knew examples of successful quit attempts by patients. The smoke-free policy was reported to have had a beneficial impact on the Trust image by 32%. Indicators to measure these impacts however were generally not defined.

Tables 2 and 3 give relevant quotes which expand on the categorisation of responses above.

**Table 2:** Interview Quotes concerning aspects of smoke-free policy implementation

**Aspect**	**Quote from Telephone Interview**
**Challenges: Extent of policy**	*"Having ambiguities, (...) having exemptions – where do you draw the line? The approach we took is: the message is "no", because if the message is, ""Well, you can do it for these patients or these patients...", it becomes confusing and leads to misinterpretations." (1)*
	*"We struggled whether we should include this *[exemptions on a case-by-case basis in distressing situations]*in the policy, in case that people start using this as an excuse to allow smoking... We decided to let people use common sense on that rather than saying "Yes, in a distressing situation"... It's very hard to measure what is a distressing situation." (2)*
**Challenges: Enforcement**	*"It *[enforcement]*is a real challenge. What we expect is every member of staff to play a part in it. (...) Obviously, they have to be careful, you know, there is the issue of violence and aggression (...) The biggest fear that our staff have is that of violence and aggression, of taking someone's habit away from them – how are they going to react?" (3)*
	*"Now, what we know is that getting patients to stop smoking outside the main entrance has been a nightmare..." (4)*
	*"We struggle at the entrance of A&E, and a little bit still in front of maternity..." (5)*
**Challenges: Litter/Smoking in adjacent public grounds**	*"This *[litter]*is a real dilemma- we took away the smoking areas that we have had, and we took away the bins, that's on the basis that if people aren't allowed to smoke they shouldn't have a place to put their fag ends – of course what people then do is drop them on the floor."(4)*
	*"The litter problem has actually shifted from the Trust to the council, because patients are going outside *[the premises]*to smoke." (6)*
	*"Smoking staff congregating in front of the premises doesn't make a good impression in terms of the Trust image... And also, it's a health and safety issue, if staff wander off and nobody knows where they are." (7)*
**Impacts**	*"You actually go outside and you can breathe fresh air (...) so I think it's a pleasurable environment for patients, I think that's the fundamental impact that it has had."(6)*
	*"We find now you walk through the hospital grounds and it's quite rare to see anyone smoke."(8)*
	*"I would say with confidence that a number of people *[staff] *have, as a result of the promotion we are doing, given up smoking, but we don't have specific figures on that."(9)*
	*"I think that, when people go to the pre-assessment where (...) smoking status is recorded and smokers are told that they can't smoke whilst they are in here, this adds to their motivation to stop smoking."(5)*

**Table 3:** Examples of Trusts' innovative Approaches

**Topic**	**Innovative Approaches to address Challenges**
**Challenges: Exemptions to policy**	*Written guidance to decide whether exemptions on a case-by-case basis can be made, has been developed for ward managers*
**Challenges: Enforcement**	Cards stating the Trusts' smoke-free policy and giving contact details of NHS stop smoking service have been developed to hand over to smokers (risk-free alternative to approaching them verbally)*
	Step-by-step guide regarding enforcement/breeches of policy for staff/visitors/patients has been drafted*
	Areas in front of A&E and Maternity have been targeted through extended signage, thorough cleaning, & challenging smokers*
**Support for Staff**	*Theatre staff have been supported through targeted support in smoking cessation in view of the "logistic" difficulties in leaving the premises to smoke*
	*A "Big Action Plan" with a focus on extended support for staff is being developed in a joint effort with the community (PCT, pharmacists)*
**Support of Patients**	Smoking status/willingness to stop smoking are assessed on admission and recorded in standard documentation*
	*A clinical pathway for smoking cessation has been developed (Mental health Trust)*
	A holistic approach towards physical well-being is taken through combined exercise/stop-smoking programs (Mental health Trust)*
	*Former smoking rooms have been transformed into recreational spaces for patients (Mental health Trust)*
**Impacts**	*Impact of the policy is going to be reviewed for a) smoking prevalence among staff before and after, b) cleanliness of premises, c) Trust image by means of repeated surveys*

* mentioned by more than one respondent

### Site Visits

Twenty-two hospital sites, covering three English regions, were chosen for site visits due to their easy accessibility to the investigator. They belonged to 15 Trusts, 10 of which were acute Trusts (covering 17 sites). Four of the Trusts (three acute, one mental health) had not responded to the questionnaire survey. Depending on the size of the premises, visits lasted around one hour.

#### Acute Sites

Of the seven acute Trusts (covering 10 sites) visited that had responded to the questionnaire, all had reported that their smoke-free policies applied across the premises including grounds. In all cases, the validity of this information was proved by signage and other means of communication (e.g. posters, leaflets) during the visits. Two of these Trusts had stated that no exemptions were allowed. However, with the exception of one site belonging to a Trust that had not responded to the survey, at all acute sites visited (94%), patients and visitors were observed smoking in the grounds, clearly in breach of the policy, and often in close proximity to signage requesting no smoking. At almost a third of sites, more than ten policy breaches were witnessed during the visits. No attempts at enforcement were observed. At six sites (35%), smokers clearly identifiable as members of staff through uniform or badges were observed to breach the policy, sometimes close to entrances. Areas that were affected by the infringements especially included the main and side entrances, and spaces in front of A&E and maternity departments. There was no evidence of smoking indoors during any of the visits.

At 13 of the acute sites visited, NHS stop smoking services were advertised, and at four sites, leaflets detailing the Trust's smoke-free policy were provided. Only six sites provided further written information on nicotine dependence/smoking cessation in publicly accessible areas. NRT products were available in 10 of the 13 pharmacies visited.

**Figure 1:**
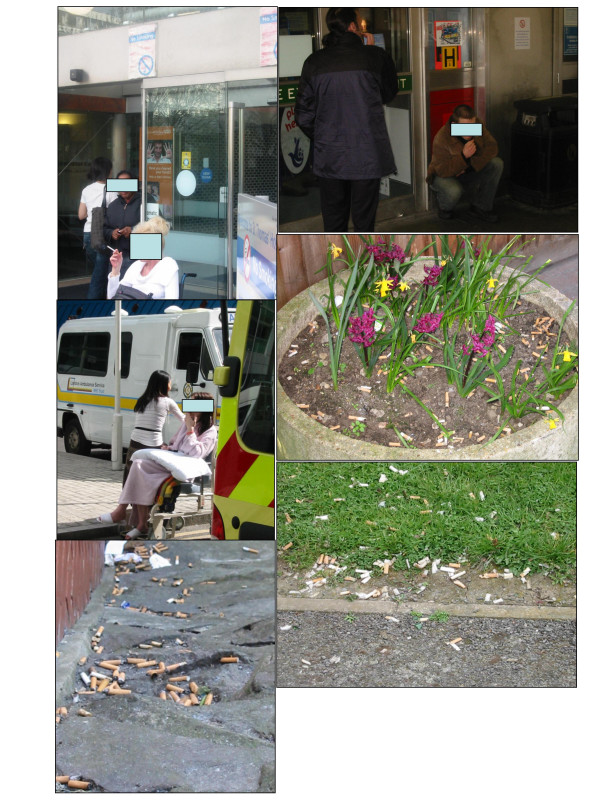
Observations at Trust Sites.

The majority of the outdoor premises were considerably polluted by cigarette ends, some of them to such an extent that the litter dominated the image of the whole site (see Figure 1).

For four of the five Trusts where more than one site was visited, variation was observed between the degree of policy promotion (signage, other information) and enforcement (breaches, litter) across the sites. For the seven Trusts that had responded to the questionnaire, deviance between the information provided and observations were largely due to smoking being tolerated on the premises (especially at entrances), which had not been declared as an exemption in the questionnaire.

#### Mental Health Settings

Despite limited insight into mental health settings due to restricted access, the observations carried out in entrance and accessible outdoor and waiting areas showed that efforts to implement smoke-free policies had been undertaken. There was little evidence of cigarette ends and policy breaches.

## Discussion

This study indicates that efforts to implement smoke-free policies had been undertaken by virtually all Trusts. However, hospitals were still not smoke-free. Exemptions were frequently granted and the site visits indicated that smoking on premises was still prevalent, even by staff in uniform.

Policy infringements in the grounds were widespread and appeared to be widely tolerated undermining the ethos of hospitals as health promoting organisations. This therefore supports findings that a main challenge of smoke-free policy implementation in hospital settings lies in its sustained enforcement [17,18]. Commonly mentioned problems included: regular policy breaches in certain areas; reluctance of staff to engage in active policy enforcement due to risks of abuse; litter of premises and adjacent grounds; and smokers congregating in front of Trusts. In the future, identifying ways to support staff in enforcing smoke-free policies effectively and without feeling at risk would appear to be useful.

Compared to the results of an earlier study [13], a considerably increased number of Trusts reported being completely smoke-free (33% compared to 10% in 2003) indicating that smoke-free grounds as well as buildings is feasible. As 80% of Trusts reported using the HDA guidance, it is possible that this increasing trend towards going completely smoke-free (the "gold standard" promoted in the guidance) might be related to the provision of written guidelines, as has been suggested previously[6].

Exemptions to the smoke-free policy, however, were reported to be granted by half of acute and 78% of mental health Trusts, most frequently for bereaved relatives in acute Trusts, and for patients in mental health settings. At the time of questionnaire completion, 3% of all acute Trusts, and 33% of all mental health settings reported providing smoking rooms. Free text comments and interviews indicated however that efforts to meet the legal demands by July 2007/2008 were currently being undertaken. There was evidence from the interviews that even if exemptions had not been reported in the questionnaire, they were often still being made on a case-by-case basis.

A systematic evaluation of the effect of smoke-free policies across the NHS is not possible as indicators to measure the impact had generally not been defined. Having a set of indicators would be useful to assess policy implementation in the future including objective measures of exposure to tobacco smoke. Nevertheless, all interviewees were convinced of the beneficial impacts of the policy, which might explain why the great majority of questionnaire respondents rated it as successful despite reported frequent infringements.

Reduced exposure to environmental tobacco smoke in the buildings and less smoking in the grounds were mentioned by over half the respondents as benefits. Further benefits referred to a possibly reduced prevalence of smoking in staff, and to the potential to inspire quitting attempts by patients, as discussed elsewhere [11]. Many respondents felt that going smoke-free had positively influenced their Trust's image of a health promoting organisation.

Constant reinforcement of the policy and its active promotion were regarded as central determinants of successful policies. Despite the emphasis respondents placed on the provision of educational material and promotional campaigns, evidence of such proved comparatively sparse during the site visits. Given only around a quarter of questionnaire respondents reported having specific budgets for smoke-free policy implementation, a shortage of financial resources may explain this.

Support for staff, both to engage in enforcement without risking abuse, and to cope with nicotine dependence during working hours were regarded important by interview respondents, the latter having been described in previous work[17,19,20]. However, there was anecdotal evidence that due to low demand for cessation from staff, offers of support had been reduced, which might ultimately be counterproductive. This finding suggests that future research would be useful to identify the type of support and delivery that staff would find most attractive. Similarly, identifying how to best motivate and support patients to stop smoking would appear important.

Many interviewees highlighted the importance of a "change in culture" after the enforcement of the Health Act 2006, which would gradually introduce a new "non-smoking norm".

### Study limitations

Legal and political requirements relating to smoke-free policies, as well as the formal request of information under the EIR may have added a small degree of reporting bias to the study. This might have led to an overreporting of the extent of policy implementation particularly in the 17% of respondents who completed questionnaires after the EIR request. The fact that study participants were largely responsible for implementing smoke-free policies in their Trust might have impaired the objectivity of responses. No information was provided by 21% of the study population, which limits the generalisability of results. However, the site visits included four non-respondent Trusts, all of which had also implemented smoke-free policies. Findings of site visits are limited to a small subsample limited to three English regions and may therefore not be generalisable. The choice of one study participant per Trust may constitute a further source of bias because the perspective was generally restricted to that of a non-clinical executive, and the information provided likely to refer predominantly to one site. Eighty per cent of Trusts comprised more than one site and policy implementation was observed to vary across different sites of the same Trusts. However, generally the information provided in the questionnaires was found to be valid in cross-checks during site visits. Deviations largely referred to the lack of policy enforcement in hospital grounds.

## Conclusion

For many years the NHS has been urged to go smoke-free and set an example to other organisations. Amongst the first public organisations to establish smoke-free environments, important lessons can be learned from their experience. This study indicates that all Trusts had implemented smoke-free policies yet smoking was still prevalent particularly around entrances. Effective enforcement is critical and more must be done to find better ways of supporting staff to engage effectively in enforcement, manage nicotine withdrawal and stop smoking on site. If smoking continues to be tolerated on NHS premises, there is a risk that the NHS will now fall behind other work and public places.

## Competing interests

The author(s) declare that they have no competing interests.

## Authors' contributions

ER conducted the survey, the telephone interviews, and the observations at Trust sites, as well as the respective analyses. She drafted the manuscript.

AMCN and JB supervised the study, supported ER in developing the study instruments, and reviewed analyses, results and the manuscript draft.

All contributing authors have read and approved the final manuscript.

## Pre-publication history

The pre-publication history for this paper can be accessed here:


